# Systemized approach to equipping medical students with naloxone: a student-driven initiative to combat the opioid crisis

**DOI:** 10.1186/s12909-024-05221-8

**Published:** 2024-03-06

**Authors:** Shahin A. Saberi, Sydney Moore, Sienna Li, Rory Vu Mather, Mary B. Daniels, Amrita Shahani, Antje Barreveld, Todd Griswold, Patrick McGuire, Hilary S. Connery

**Affiliations:** 1 Harvard Medical School, Boston, MA USA; 2 Harvard/MIT MD-PhD Program, Boston, MA 02115 USA; 3 Department of Anesthesia, Critical Care & Pain Medicine, Massachusetts General Hospital, Boston, MA USA; 4 Program in Health, Sciences, and Technology, Massachusetts Institute of Technology, Boston, MA USA; 5 Outpatient Pharmacy, Brigham and Women’s Hospital, Boston, MA USA; 6 Department of Anesthesiology, Newton-Wellesley Hospital, Newton, MA USA; 7 Center for Mindfulness and Compassion, Cambridge Health Alliance, Cambridge, MA USA; 8 Department of Psychiatry, Brigham and Women’s Hospital, Boston, MA USA; 9 Division of Alcohol, Drugs, and Addiction, McLean Hospital, Belmont, MA USA

**Keywords:** Naloxone, Opioid overdose crisis, Public health, Opioid overdose prevention

## Abstract

**Background:**

Naloxone is an effective and safe opioid reversal medication now approved by the U.S. Food and Drug Administration (FDA) for use with or without a prescription. Despite this, naloxone dissemination lags at a time when U.S. opioid-related mortality expands. The authors proposed distributing naloxone to all U.S. medical students using established statewide standing prescription orders for naloxone, eliminating the financial burden of over-the-counter costs on students and streamlining workflow for the pharmacy. By focusing naloxone distribution on medical students, we are able to capitalize on a group that is already primed on healthcare intervention, while also working to combat stigma in the emerging physician workforce.

**Methods:**

Beginning August 2022, the authors established a partnership between Harvard Medical School (HMS) and the outpatient pharmacy at Brigham and Women’s Hospital (BWH) to facilitate access to naloxone for HMS medical students. BWH developed a HIPAA-secure electronic form to collect individual prescription information. BWH pharmacists processed submissions daily, integrating the naloxone prescription requests into their workflow for in-person pick-up or mail-order delivery. The electronic form was disseminated to medical students through a required longitudinal addiction medicine curriculum, listserv messaging, and an extracurricular harm reduction workshop.

**Results:**

Over the 2022–2023 academic year, 63 medical students obtained naloxone kits (two doses per kit) through this collaboration.

**Conclusions:**

We propose that medical schools advocate for a hospital pharmacy-initiated workflow focused on convenience and accessibility to expand naloxone access to medical students as a strategy to strengthen the U.S. emergency response and prevention efforts aimed at reducing opioid-related morbidity and mortality. Expansion of our program to BWH internal medicine residents increased our distribution to over 110 healthcare workers, and efforts to expand the program to other BWH training programs and clinical sites such as the emergency department and outpatient infectious disease clinics are underway. With more than 90,000 medical students in the U.S., we believe that widespread implementation of targeted naloxone training and distribution to this population is an accessible approach to combating the public health crisis of opioid-related overdoses.

## Background

In 2020, opioid-related deaths increased to over 85,000 per year, with over one million lives lost due to opioid overdose in the past three decades [1]. Naloxone, an opioid receptor antagonist that can be administered intranasally or intramuscularly to reverse overdoses outside of medical settings, has been shown to decrease overdose-related mortality among people who use opioids [2]. 

In 2018, the U.S. Surgeon General identified potential bystanders, including all community members, as a crucial population for naloxone distribution [3]. However, the distribution and availability of naloxone to the general public is controlled on a state-by-state basis, with no federal standing order that would mandate access to naloxone and allow pharmacies to dispense without a prescription [4]. 

In July 2020, the Massachusetts Department of Public Health issued a statewide standing order that allowed retail pharmacies to dispense naloxone without a prescription and required them to maintain a continuous, sufficient supply to meet the needs of the community [5]. After the standing order took effect, a statewide Massachusetts purchase trial demonstrated high levels of naloxone stocking and dispensing in retail pharmacies, and an analysis of pharmacy reports showed increases in both the quantities of naloxone dispensed and the number of pharmacies dispensing naloxone [6, 7]. 

Subsequently, the U.S. Food and Drug Administration (FDA) approved naloxone (4 mg, intranasal spray) for over-the-counter (OTC) use in March 2023. Importantly, OTC medications are not covered by insurance and the out-of-pocket cost could still pose a significant barrier to low-income individuals. Additionally, a prescription will still be required to obtain other formulations and doses of naloxone designed for community-based reversal (e.g., 8 mg intranasal spray). Thus, while this approval paves the way for naloxone to be sold directly to potential bystanders without a prescription in commonplace settings such as grocery or convenience stores, barriers to access will likely persist.

Recent studies have indicated that increasing availability and distribution of naloxone to laypeople and first responders could prevent up to 21% of opioid overdose deaths in the United States [8]. Unfortunately, high rates of opioid-related overdose deaths continue to persist, and the need for widespread distribution of naloxone into the hands of people who are likely to act is paramount. Medical students, as the target of this intervention, make up an ongoing audience of future healthcare professionals who will provide addiction care to patients with SUDs. More than 90% of Massachusetts medical students strongly agree that it is important to know how to treat substance use disorder (SUD) as a physician; however, the majority did not feel that they were adequately trained to manage patients with SUDs [9]. Furthermore, bias against patients with SUDs exist from both students and practicing physicians. Studies have shown that physicians with stigmatizing attitudes are less likely to prescribe medications to treat SUD, despite the strong evidence for their efficacy [10, 11]. 

 The Substance Use and Pain Curriculum Committee was established in 2016 at Harvard Medical School (HMS) as part of a student-led effort to advocate for and further develop an addiction and pain management curriculum. In June 2022, students of the committee called the four retail pharmacies within a 0.5-mile radius of HMS to try to obtain a bulk number of doses of naloxone to distribute to the entire medical school through the Massachusetts statewide standing order. However, we learned that health insurance coverage is only applicable for personal naloxone reversal use, which requires the person to have an opioid-related diagnosis for approval and would not allow for distribution to our peers. The out-of-pocket cost without insurance would be $90/kit (2 nasal naloxone dispensed per kit). Additionally, a $300 Massachusetts Controlled Substance Registration (MCSR) would be required to distribute naloxone to students, bringing the total cost to equip a first-year class of ~ 200 Harvard Medical students to >$15,000 (MA General Law Ch. 94 C, Sect. 7). Although the recent FDA approval of OTC naloxone is a great achievement to increase accessibility to naloxone, bypassing insurance coverage renders cost barriers for students with significant loan debt and high cost of living, and inadvertently introduces a disincentive to naloxone dissemination. We sought to create a financially feasible option to supply HMS medical students with naloxone.

Our intervention, which teaches students about SUD management and treatment at the beginning of their medical education, is aimed at supporting future generations of physicians graduating with the confidence, empathy, and clinical knowledge to care for patients experiencing SUD. By placing naloxone in the hands of trained medical students, we hope to reduce stigma, normalize the carrying of naloxone, provide a tangible intervention that reflects the student’s responsibility as a community member and future medical professional, and empower students to respond and address overdose. Moreover, medical students must be knowledgeable about and comfortable using naloxone in order to properly use it in clinical settings, prescribe it to patients with SUDs, and confidently train patients in its use. We designed our workflow to capitalize on existing connections between medical schools and their affiliated hospitals, making the basic framework easily customizable and generalizable to medical schools across the country. With more than 90,000 medical students in the U.S., we believe that widespread implementation of targeted naloxone training and distribution to this population is an accessible approach to combating the public health crisis of opioid-related overdoses.

## Methods

### Design and intervention

**Fig. 1:**
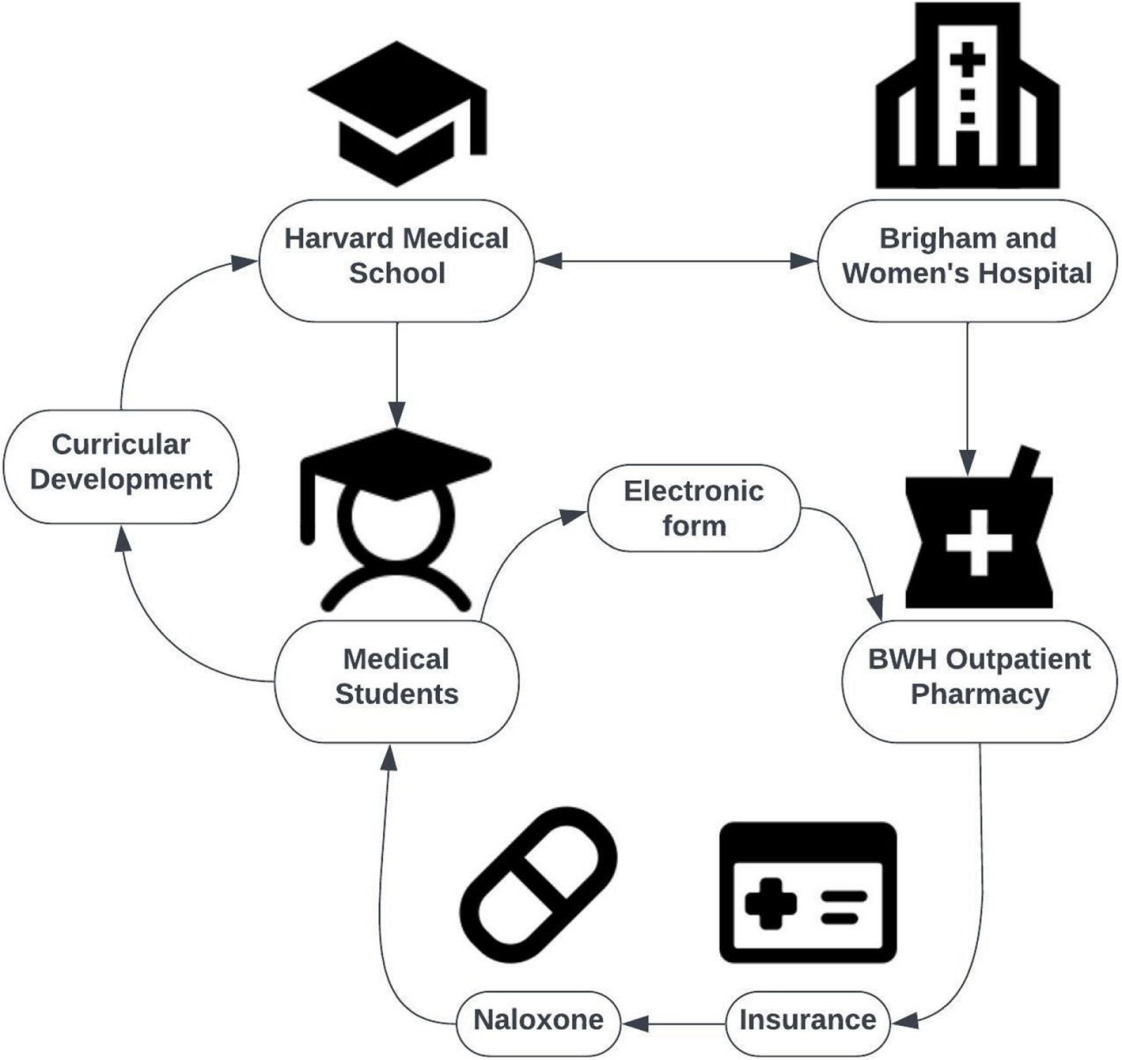
Workflow for naloxone distribution to medical students. By establishing a connection with an outpatient pharmacy of a Harvard Medical School-affiliated hospital, medical students were able to access Naloxone by registering through the hospital network as employees/trainees. BWH = Brigham and Women’s Hospital (Boston, MA)

In August 2022, we conducted a prospective single-arm intervention pre-post study to evaluate the efficacy of implementing a streamlined system in which HMS medical students use the Massachusetts statewide standing order to obtain naloxone from the outpatient pharmacy at Brigham and Women’s Hospital (BWH) (Fig. 1).

We confirmed the feasibility of the naloxone procurement process with the BWH outpatient pharmacy and collaborated with the Manager of Outpatient Pharmacy Services at BWH outpatient pharmacy to develop a streamlined system for students to place orders for naloxone. The BWH outpatient pharmacy created a Microsoft Teams electronic form using institutional accounts to ensure HIPAA compliance (Table 1). Microsoft Teams allowed us to easily create a user-friendly form that is also frequently used as a HIPAA-compliant platform at BWH.

The form requires a name, date of birth, insurance information, phone number, and email for notification purposes. The form also contains options for obtaining the prescription, including in-person pick-up or mail order. If mail order is requested, submitters must provide a mailing address. To complete the form, an individual must be registered through the BWH network either as an employee/trainee or as a patient, as Massachusetts requires that one be either an employee or patient of the hospital when they request a prescription at that hospital’s outpatient pharmacy. Medical students who are not registered through the BWH network must register with patient registration. Only one form per participant is allowed and the email address that the participant uses on the form is used to confirm that no duplicate orders are placed.

Every 24 h, BWH outpatient pharmacists check for new form submissions. As new submissions are received, pharmacists then process the prescription using the standing statewide order for naloxone. These prescriptions are integrated into the typical pharmacy workflow, with in-person pick-up orders waiting on the shelf and mail-order deliveries delivered through the preferred mail carrier. The expected turnaround time was two to three business days.

We include a disclaimer that the requester was attesting to billing their prescription to their insurance, and if there was a copay, this would be the requestor’s responsibility. HMS student health insurance, as well as most health insurances, ensures a $0 copay. We also include a YouTube video that describes how to use naloxone nasal spray (https://youtu.be/LmxZkNW7VKM).

The form is publicized at several points during the four-year medical curriculum. First-year medical students are required to complete Basic Life Support (BLS) training, which, at HMS, includes a five-minute naloxone training [12]. Naloxone training within the BLS course serves as the first formal introduction to opioid overdose management at HMS and allows students to understand the basics of identifying potential opioid overdose. Students are encouraged to procure naloxone at this stage using our form, equipping students to take immediate action in instances of opioid overdose early in their medical training.

Naloxone procurement information was also incorporated into lectures relevant to substance use found in the first- and second-year preclinical curriculum, bridging teachings about the physiology and epidemiology of opioid use with clinical intervention tools. Lecturers were provided with a slide to add to the end of the lecturers containing a QR code to access the naloxone procurement form. Students are again offered the opportunity to obtain naloxone just prior to beginning their clinical clerkships during a didactic lecture on the treatment of substance use disorders. Finally, we incorporated naloxone distribution into a pre-graduate practicum for fourth-year medical students that teaches medication-based treatments for opioid use disorder, which aims to equip medical trainees with naloxone as they transition to the next stage of their medical training.

Beyond core curricular integration, we also invite students to complete the form via HMS distribution listservs and at the end of a voluntary extracurricular event that educates attendees on harm reduction techniques, including the use of naloxone [13]. 

### Participants and setting

The primary audience of the intervention included the estimated 540 medical students in the Pathways program at Harvard Medical School. All students who participated did so on a voluntary basis.

### Evaluation

Efficacy of the intervention was evaluated by measuring the number of HMS medical students who received naloxone from the BWH outpatient pharmacy using the HIPAA-secure electronic form.

**Table 1:** Requested information on the electronic form for naloxone distribution

Requested Information	Response Options
Full legal name	Free text field
Phone number	Free text field
Email	Free text field
Date of birth	mm/dd/yyyy
Do we have permission to bill your insurance for nasal naloxone?	◦ Yes ◦ No
You will not be charged for naloxone at the time, but this is permission to bill your insurance at the time of request. Note that you may still have a remaining copay to pay.	
If you know your Prescription Insurance, please provide the following information: Bin: PCN: Group: ID/Subscriber #:	Free text field
How would you like to pick-up your prescription? Mail Order is free, but make sure to include your address in the “Other” field.	• Outpatient Pharmacy @ 45 Francis St, Boston • Mail Order - please leaving mailing address in Other Field • Outpatient Pharmacy @ 20 Patriot Place, Foxborough • Outpatient Pharmacy @ 850 Boylston St, Chestnut Hill • Other - ______________
Additional comment	Free text field

## Results

We successfully integrated opportunities for naloxone procurement into five parts of the HMS Pathways curriculum including BLS training, first- and second-year lectures on the topic of substance use, a pre-clinical didactic addressing substance use treatment, and again in a pre-graduate practicum on medication-based treatment for substance use disorders for fourth-year students. The creation and implementation of the curricular changes was accomplished over nine student meetings, four additional meetings with supervising staff, and two virtual meetings with the BWH pharmacy manager. Since initiating this program in August 2022, 63 of 540 total Harvard medical students have procured a naloxone kit (contains two doses) using this form as of January 2024. 30 of the 63 students were recruited by the extracurricular event on harm reduction. All orders for naloxone were filled by the BWH outpatient pharmacy.

## Discussion

Over the past five decades, naloxone has progressed from an experimental opioid antagonist to the standard of care for reversing opioid overdoses. While medical schools across the U.S. are increasingly incorporating substance use disorder curricula, including training on opioid overdose prevention and response, access to naloxone for medical students remains inconsistent [12, 14, 15]. Our work presents an efficient method to expand naloxone access to medical students, the next generation of healthcare workers.

Medical students who undergo opioid overdose prevention and response training may still harbor uncertainty about the legal aspects of naloxone access [14]. Additionally, the stigma surrounding naloxone among healthcare workers poses a significant barrier to its distribution [16–19]. By providing naloxone to medical students during their training, our initiative aims to address these uncertainties and negative biases, ensuring that students are equipped to respond effectively to opioid overdoses throughout their medical careers.

Medical students can play a crucial role in opioid overdose response and prevention, as they spend significant amounts of time with patients and are often involved in volunteer clinics that serve patients who may experience substance use disorders [14]. By targeting medical students, our intervention ensures that future clinicians are empowered to educate patients about overdose risk management and naloxone accessibility, regardless of their intended specialty.

Our system of naloxone distribution relies upon established relationships between teaching hospital pharmacies and the associated medical schools, making it easily replicable across medical institutions with similar partnerships. While our initiative was spearheaded by medical student leadership within the substance use and pain curriculum at our institution, we recognize that not all medical schools will have a similar framework in place. Therefore, we propose alternative strategies for integrating naloxone training and procurement into medical school curricula, tailored to the unique needs and resources of each institution. For example, BLS training is mandatory for first-year U.S medical students. Integration of naloxone training in BLS training can provide an early and concrete introduction to the topic of substance use. Moreover, although formats and placement may vary, most medical schools address substance use in some capacity. Information about naloxone procurement can be integrated into lectures or modules with as much or as little expansion as is practical. A more involved option includes student-led advocacy for curriculum reform as it relates to education on opioid use disorder diagnosis and management. Finally, we found that widespread electronic form distribution using listserv messaging effectively communicated with medical students across all years.

We believe that our method of naloxone distribution can be feasibly scaled up to include all healthcare training programs within a given healthcare system. Our ongoing efforts include extending naloxone distribution to all BWH internal medicine residents, which has increased our total number of distributed naloxone kits to 110 healthcare workers. In addition to our efforts with providers, we are exploring ways to extend our workflow to patient-facing forms in ambulatory clinics. For instance, our team is currently collaborating with emergency departments and infectious disease clinics, as they serve a higher proportion of patients experiencing substance use disorder. Additionally, we aim to establish regularly scheduled in-hospital booths staffed by pharmacists to provide a streamlined process for patients to place naloxone prescriptions in under five minutes.

Interestingly, opioid overdose response and prevention training programs promote increased knowledge and positive attitudes toward patients with substance use disorders [20]. While we did not collect data on medical student attitudes or demographics related to naloxone procurement to minimize friction in the process and protect individual privacy, future research could explore changes in attitudes with ongoing naloxone distribution efforts. Findings of reduced biases toward patients with substance use disorders and naloxone utilization could underscore the importance of scaling this initiative to other clinical training environments.

Our data indicate a notable uptake among students, with 63 of 540 medical students obtaining naloxone in the year since the initiation of our initiative. Nonetheless, we recognize the importance of optimizing this uptake to ensure broader coverage within the student body. Moving forward, we plan to implement targeted interventions to enhance awareness and encourage naloxone procurement among students through social media campaigns, in-person presentations at orientation events, and targeted emails following specific points of the curriculum. In future iterations of the program, we aim to enhance the accessibility and convenience of naloxone procurement by exploring alternative distribution methods, such as on-campus distribution points following substance use disorder curricular events.

In summary, we present a workflow for how to equip medical students with prescription naloxone, driven by the collaborative efforts of medical students, school administrators, pharmacists, and physicians. As the landscape of naloxone regulation evolves, it is possible that future initiatives will need to adapt to policies that regulate the drug’s distribution, marketing, and administration. By empowering medical students with naloxone, we enhance the emergency response system for opioid overdoses and cultivate a future generation of healthcare workers who will be advocates for persons who use substances.

## Conclusions

In this work, we propose that medical schools advocate for a hospital pharmacy-initiated workflow focused on convenience and accessibility to expand naloxone access to medical students, hospital staff, and other trainees, as a strategy to strengthen the U.S. emergency response and prevention efforts aimed at reducing opioid-related morbidity and mortality. 63 kits of naloxone have been distributed to HMS medical students in just over a year, and the existing framework is being used to further expand the reach of distribution to both healthcare workers and patients. By targeting naloxone teaching and distribution to medical students, a community that is already being trained in clinical interventions, our goal is to arm more people with naloxone who are already primed and prepared to act in an emergency. With more than 90,000 medical students in the U.S., we believe that widespread implementation of targeted naloxone training and distribution to this population is an accessible approach to combating the public health crisis of opioid-related overdoses.

## Data Availability

Our work does not contain any data that was analyzed in order to establish this naloxone distribution process for medical students. All forms and materials used to establish this naloxone procurement process are available from the corresponding author upon request.

## Abbreviations

HMS: Harvard Medical School
BWH: Brigham and Women’s Hospital
SUD: Substance Use Disorder
MCSR: Massachusetts Controlled Substance Registration
FDA: Food and Drug Administration
